# Low plasma vitamin D is associated with increased 28-day mortality and worse clinical outcomes in critically ill patients

**DOI:** 10.1186/s40795-023-00801-1

**Published:** 2024-01-09

**Authors:** Fatemeh Sistanian, Alireza Sedaghat, Mohaddeseh Badpeyma, Majid Khadem Rezaiyan, Ahmad Bagheri Moghaddam, Golnaz Ranjbar, Mostafa Arabi, Mohammad Bagherniya, Abdolreza Norouzy

**Affiliations:** 1 Department of Nutrition, Faculty of Medicine, Mashhad University of Medical Sciences, Mashhad, 985138002421 Iran; 2 Department of Anesthesiology, Faculty of Medicine, Lung Diseases Research Center, Mashhad University of Medical Science, Mashhad, Iran; 3 Student Research Committee, Tabriz University of Medical Sciences, Tabriz, Iran; 4 Department of Clinical Nutrition, Nutrition Research Center, School of Nutrition and Food Sciences, Tabriz University of Medical Sciences, Tabriz, Iran; 5 Department of Community Medicine, Faculty of Medicine, Mashhad University of Medical Sciences, Mashhad, Iran; 6 Department of Basic Sciences, School of Medicine, Neyshabur University of Medical Sciences, Neyshabur, Iran; 7 Nutrition and Food Security Research Center, Department of Community Nutrition, School of Nutrition and Food Science, Isfahan University of Medical Sciences, Isfahan, Iran; 8 Anesthesia and Critical Care Research Center, Isfahan University of Medical Sciences, Isfahan, Iran; 9 Metabolic Syndrome Research Center, Mashhad University of Medical Sciences, Mashhad, Iran

**Keywords:** Critically Ill patients, Vitamin D Deficiency, Intensive care unit, Mortality, Vitamin D

## Abstract

**Background & objective:**

Patients in the intensive care unit have a high prevalence of vitamin D deficiency (VDD). In the present study, clinical outcomes in the ICU were analyzed with vitamin D status.

**Materials and methods:**

In this prospective, multicenter study, sampling was conducted on seven ICUs in three hospitals. Within the first 24 h of ICU admission, patient’s serum vitamin D levels were measured, and their disease severity was monitored using the scores of acute physiologic assessment and chronic health evaluation II (APACHE II), sequential organ failure assessment (SOFA), and the modified Nutrition Risk in Critically ill (mNUTRIC) score.

**Results:**

A total of 236 patients were enrolled in this study, of which 163 (69.1%) had lower vitamin D levels than 20 ng/ml upon ICU admission. The patients with VDD had higher APACHE II scores)*P* = 0.02), SOFA scores (*P* < 0.001), and mNUTRIC scores (*P* = 0.01). Patients with sufficient levels of vitamin D (> 30 ng/ml) had a shorter stay at ICU (*P* < 0.001). VDD was independently associated with 28-day mortality (OR: 4.83; 95% CI: 1.63–14.27; *P* = 0.004).

**Conclusion:**

The data showed that VDD was common among the critically ill and was related to a more severe course of illness and a higher mortality rate.

**Supplementary Information:**

The online version contains supplementary material available at 10.1186/s40795-023-00801-1.

## Introduction

Vitamin D is a fat-soluble prohormone synthesized in the skin in response to sunlight exposure or is received in small amounts through dietary intake [1]. Vitamin D is involved in the local immune responses to pathogens and the inflammatory pathways of systemic infections. Vitamin D deficiency (VDD) is associated with an increased risk of sepsis in critically ill patients [2, 3]. According to the literature, normal vitamin D levels are defined based on serum cholecalciferol levels higher than 30 ng/ml, while lower serum levels than 30 ng/l represent vitamin D insufficiency; deficiency generally refers to lower levels serum levels than 20 ng/l [4]. Generally, evidence indicates that VDD is a major nutritional challenge for Iranian authorities, as the prevalence of VDD in Iran's population has been reported to be up to 98% [5, 6].

Serum vitamin D levels in healthy individuals depend on several factors, including diet, sunlight exposure, body fat percentage, and melanin content in the skin. Typically, critically ill patients have several problems and comorbidities, such as acute pancreatitis, multiple organ failure, critically ill obesity, surgical procedure, trauma, and sepsis. In these patients, immobility, fluid retention, inflammation, and renal conditions deteriorate their condition and cause deficiency more frequently than in the average population [7, 8]. The function of clinical nutrition in alleviating and managing the morbidities of patients is crucial [9]. In the critical care setting, VDD is associated with adverse outcomes such as infections, increased length of hospital stay, acute kidney injury, and higher mortality [10, 11]. Vitamin D plays a vital role in the immune system through its receptors on various immune cells, including activated CD4 and CD8 T cells, B cells, macrophages, neutrophils, and dendritic cells. Vitamin D also regulates the production of immunoglobulins [12].

VDD in patients with acute conditions is associated with infections, the progression of sepsis and acute respiratory distress syndrome, and increased mortality [3]. The association between vitamin D concentration and ICU outcomes was also previously assessed in some studies [13, 14]. However, the role of vitamin D in critically ill patients remains unclear [15]; thus, a multicenter study with a high number of patients compared to prior samples may provide reliable results for further investigation. In addition, it is still unknown whether VDD in patients admitted to the intensive care unit (ICU) indicates the severity of the disease or is a significant cause of mortality with direct effects [16]. The current study set out to examine the correlations between plasma vitamin D levels, clinical outcomes, and mortality in 236 patients admitted to the ICU.

## Materials and methods

This prospective, observational study was performed in four medical, one surgical, and two trauma ICUs in Mashhad, Iran, during July 2019**-**March 2020. A convenience sample of the critically ill patients admitted to the ICU was enrolled. The inclusion criterion of the study was the age of more than 18 years, and the exclusion criteria were (i) pregnant women, (ii) receiving 50,000 units of vitamin D 2–4 months before ICU admission, and (iii) receiving multivitamin supplements during/before the ICU admission.

Venous blood samples were collected from the patients within 24 h after ICU admission to measure the plasma levels of 25-hydroxyvitamin D. Plasma samples were collected and preserved at -80°C until the measurements. The second-generation electrochemiluminescence technology platform assessed 25(OH) D, and the results were expressed as ng/ml.

In compliance with the guidelines of the Institute of Medicine, Food, and Nutrition Board [4], the patients were stratified into three groups based on their serum 25(OH)D levels, as follows:

1. Normal group with vitamin D concentrations of > 30 ng/ml (75 nmol/l);
2. Insufficient group with vitamin D concentrations of 20–30 ng/ml (50–75 nmol/l);
3. Deficient group with vitamin D concentrations of < 20 ng/ml (50 nmol/l).

For the assessment of the severity of illness and mortality prediction in the ICUs, acute physiologic assessment and chronic health evaluation (APACHE II) (Additional file 1.docx: Table S1) and sequential organ failure assessment (SOFA) (Additional file 1.docx: Table S2) was applied. APACHE ӀӀ score included variables such as mean blood pressure, heart rate, temperature, respiratory rate and Glasgow Coma Score, white blood cell count, hematocrit, potassium, sodium, creatinine, serum Pa02 and pH, severe failure organs, and age [17].

The SOFA score is based on the degree of dysfunction of six organ systems, including the respiratory, liver, cardiovascular, coagulation, renal, and central nervous systems [18].

The NUTRIC, or the Nutrition Risk in the Critically Ill Score, is a helpful tool for assessing nutritional risk in ICU patients. This Score included six variables including age, number of comorbidities, APACHE II score, SOFA score, serum interleukin 6 (IL-6) concentration, and number of days in the hospital before admission to the ICU. Nevertheless, IL-6, an inflammatory marker, is not commonly measured in the ICUs, so a modified NUTRIC Score (mNUTRIC) without IL-6 (Additional file 1.docx: Table S3) was used. Patients with a score ≥ 5 were considered at high nutritional risk [19].

The collected data was included demographic characteristics (age, gender), comorbidities, cause of admission, ideal body weight, mechanical ventilation, and length of ICU stay.

APACHE II and mNUTRIC questionnaires were filled out within 24 h after ICU admission.

SOFA score was calculated every two days until ICU discharge or for a maximum of 7 days.

Anthropometric assessment, including weight (There are several methods to calculate the ideal weight. One of the best and easiest methods is to use height and the difference of 100 for men and 102 for women) [20], height (via the ulna length), and body mass index, were measured at baseline.

In addition, these patients' mid-arm circumference (MAC) was measured using an inelastic meter with an accuracy of 0.5 cm.

The mortality rate was asked by phone within 28 days of the start of the study.

### Sample size

The sample size was calculated in accordance with a previous study [13], and the length of ICU stay was used as the main variable. Based on the formula of comparing quantitative variables in two groups for the length of ICU stay and the Type I error of 5% (α = 0.05) and Type II error of 20% (β = 0.20; the power of the study was 80%) and with a 15% probability of drop-out patients during the study, the total sample size was estimated about 236 patients.

$$n=\frac{{\left(Z1-\frac{a}{2}+Z1-\beta \right)}^{2}\left({\sigma }_{2}^{2}\right)}{{\left({\mu }_{1}-{\mu }_{2}\right)}^{2}}$$

### Ethical considerations

The Ethics Committee of Mashhad University of Medical Sciences approved this work with the code: IR.MUMS.MEDICAL.REC.1398.086. Written informed consent was obtained from all patients before participating in the study. However, since some ICU patients were severely ill or unconscious, their close relatives were asked to fill out the informed consent forms before beginning the study.

### Statistical analysis

Data analysis was performed in SPSS version 11.5 (SPSS Inc., IBM Company, Chicago, IL). The normality of the distribution of variables was evaluated using the Kolmogorov–Smirnov test, and normally distributed variables were expressed as mean and standard deviation [21]. The qualitative data were reported as frequency and percentages. Variables with abnormal distribution were expressed as median (25th quartile, 75th quartile). The correlation of each variable with the outcome categories (normal, insufficiency, and vitamin D deficiency) was evaluated separately using χ^2^ for the categorical variables and the analysis of variance (ANOVA) with post-hoc and Kruskal–Wallis tests for the continuous variables. To determine the combination of the predictive variables for the optimal predictive model, univariate and multivariate binary logistic regression analyses were carried out. The multivariate analysis results were presented as a mean difference with 95% confidence intervals (CI). A value of *P* less than 0.05 was used as statistically significant.

## Results

### Patients and outcomes

**Fig. 1:**
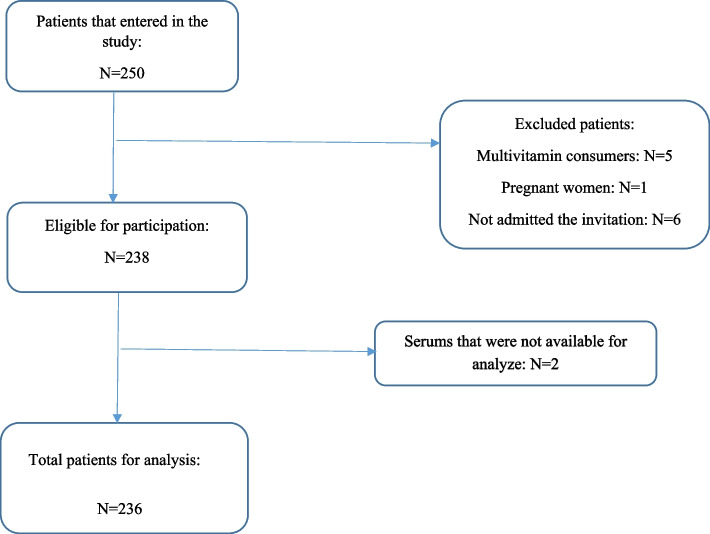
Flow diagram of study selection

In this study, 250 critically ill patients were enrolled, but the statistical analysis was conducted with 236 participants (150 males and 86 females) (Fig. 1). The baseline characteristics of patients are presented in Table 1.

**Table 1:** Comparison of Demographic and Clinical Data of Patients Based on Vitamin D Levels (*n* = 236)

Vitamin D Levels						
**Characteristics**		**Total**	**Deficient** (< 20 ng/ml) (*n* = 163; 69.1%)	**Insufficient** (20–30 ng/ml) (*n* = 34; 14.4%)	**Healthy** (30–100 ng/ml) (*n* = 39; 16.5%)	***P*-value**
Age (mean ± SD; year)		44.74 ± 16.95	45.73 ± 17.11	38.08 ± 15.99	46.41 ± 16.95	0.04^a^
Gender	Male (n %)	150 (63.6)	104 (63.8)	24 (70.6)	22 (56 .4)	0.45^b^
	Female (n %)	86 (36.4)	59 (36. 2)	10 (29.4)	17 (43.6)	
Body Mass Index (mean ± SD; kg/m^2^)		23.68 ± 1.02	23.69 ± 1.00	23.80 ± 1.05	23.53 ± 1.09	0.51^a^
APACHE II Score (day 1; mean ± SD)		14.67 ± 6.31	15.41 ± 6.49	13.02 ± 4.85	13.02 ± 6.26	0.02^a^
SOFA (1) Score (mean ± SD)		6.13 ± 3.06	6.77 ± 2.86	4.93 ± 2.67	4.50 ± 3.31	< 0.001^a^
SOFA (3) Score (mean ± SD)		6.79 ± 2.99	7.25 ± 2.85	5.07 ± 2.28	4.48 ± 3.50	< 0.001^a^
SOFA (7) Score (mean ± SD)		7.34 ± 3.16	7.79 ± 3.01	6.00 ± 2.28	5.83 ± 4.17	0.004^a^
mNUTRIC Score (mean ± SD)		2.44 ± 1.95	2.76 ± 1.96	1.70 ± 1.50	1.76 ± 1.95	0.001^a^
MAC Percentage (mean ± SD)		28.52 ± 3.24	28.65 ± 3.44	29.00 ± 2.57	27.57 ± 2.73	0.11^a^
eGFR (1)^d^ (mean ± SD; ml/min per 1.73 m^2^)		81.19 ± 34.88	79.04 ± 33.90	97.39 ± 36.77	76.40 ± 34.39	0.01^a^
eGFR (2)^e^ (mean ± SD; ml/min per 1.73 m^2^)		74.89 ± 32.89	67.85 ± 28.64	100.69 ± 36.75	83.65 ± 34.73	< 0.001^a^
Length of Ventilation (mean ± SD; day)		4.87 ± 2.91	5.62 ± 2.64	3.64 ± 3.25	2.82 ± 3.05	0.01^a^
Length of ICU stay (median (IQR); day)		9 (5–20)	10 (7–23)	9 (5–13)	6 (3–11)	< 0.001^c^
Hemoglobin (mean ± SD; g/dl)		12.36 ± 2.82	12.34 ± 2.9	12.70 ± 2.16	12.15 ± 2.82	0.71^a^
Bs (mean ± SD; mg/dl)		147.31 ± 74.19	153.80 ± 82.39	134.19 ± 54.60	137.09 ± 56.93	0.27^a^
28-day mortality						
n (%)		68 (28.8)	58 (35.6)	6 (17.6)	4 (10.3)	0.002^b^
**Cause of Admissionn (%)**						
Respiratory Disease 26 (11.1%)						
Trauma 69 (29.4%)						
Postoperative 23 (9.8%)						
Poisoning 22 (9.4%)						
Cancer 10 (4.3%)						
Myocardial Infarction 10 (4.3%)						
Neurological Diseases 55 (23.4%)						
Other 20 (8.5%)						

*APACHE II* Acute physiologic assessment and chronic health evaluation II, *SOFA* Sequential organ failure assessment, *ICU* Intensive care unit, *MAC* Mid-arm circumference, *SD* Standard deviation

^a^Analysis of variance test was used

^b^χ2 test was used

^c^Kruskal-Wallis H test was used

^d^GFR (1): At the beginning of hospitalization

^e^GFR (2): The seventh day of hospitalization

The mean age of the patients was 44.74 ± 16 years, and the age comparison between deficient (45.73 ± 17.11), insufficient (38.08 ± 15.99), and normal vitamin D (46.41 ± 16.95) levels showed a significant difference (*P* = 0.04).

The APACHEӀӀ, SOFA, and mNUTRIC scores at baseline were compared between the three groups of VDD, vitamin D insufficiency, and normal vitamin D. According to the obtained results, the severity of illness scores, including APACHE II score (*P* = 0.02) and SOFA score (*P* < 0.001) were significantly higher in patients with VDD (15.41 ± 6.49 and 6.77 ± 2.86, respectively) compared with insufficient (13.02 ± 4.85 and 4.93 ± 2.67, respectively), and normal vitamin D (13.02 ± 6.26 and 4.50 ± 3.31, respectively). Also, the mNUTRIC score comparison between the three groups (VDD: 2.76 ± 1.96, insufficient: 1.70 ± 1.50, normal: 1.76 ± 1.95) showed that the patients with VDD had higher nutritional risk (*P* = 0.01).

In addition, the mean duration of mechanical ventilation time (day) (*P* = 0.01), prolonged ICU stay (*P* < 0.001), and eGFR (*P* < 0.05) were significantly higher in the patients with VDD compared to vitamin D insufficiency and the normal group (Table 1).

**Fig. 2:**
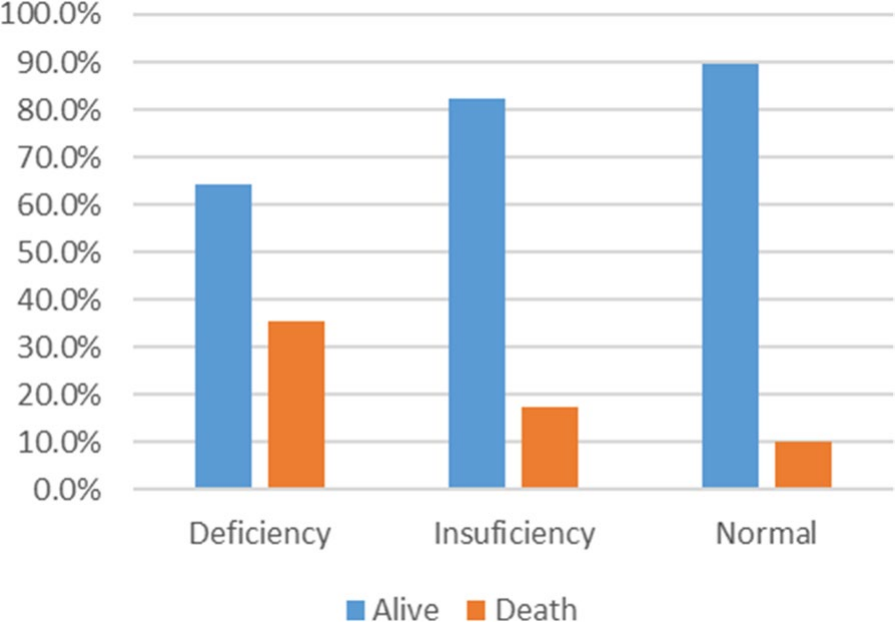
Comparison of Mortality Rates in Three Groups of VDD, Vitamin D Insufficiency, and Normal Vitamin D

All-cause 28-day mortality was estimated at 35.6% in patients with VDD, while 17.6% and 10.3% were in the vitamin D insufficiency and normal groups, respectively, and this relation was significant (*P* = 0.002) (Fig. 2).

Using binary multivariate logistic regression, the significant outcome predictor was determined. It contained severity rating and vitamin D concentration. According to the findings, VDD increased the risk of 28-day mortality by 4.83 folds compared to normal vitamin D levels. Analyses of logistic regression revealed that VDD was an independent predictor of 28-day mortality. (OR: 4.83; 95% CI: 1.63–14.27) (Table 2).

**Table 2:** Multivariate analysis of mortality predictors

Predictor	Exp. (B; Confidence Interval)	*P*-value
APACHE II Score	1.19 (1.12 ± 1.26)	0.01
Vitamin D Deficiency	4.28 (1.30 ± 14.03)	0.01
Vitamin D Insufficiency	2.37 (0.54 ± 10.36)	0.24
Normal Vitamin D (reference)	_	_

*APACHEII* Acute physiologic assessment and chronic health evaluation II

## Discussion

This present study was conducted to assess the correlation between vitamin D concentration and clinical outcomes in 236 ICU patients. Our investigation revealed that 69.1% ICU-admitted patients had low 25-hydroxy D levels at baseline. In addition, it is shown that VDD had a considerable correlation with APACHE II, SOFA, and mNUTRIC scores. Finally, vitamin D level on admission had a notable association with the 28-day mortality rate. All of these findings indicated that early evaluation of vitamin D in critically ill patients and also supplementation with vitamin D might have beneficial effects on these patients and may reduce the rate of 28-day mortality among ICU patients.

The results of this study exhibited that 69.1% of the patients admitted to the ICU had 25-hydroxy VDD, which is a higher rate in comparison to the reports of the previous studies in other countries (20–40%) [22–24]. Meanwhile, 80% of participants in Arnson' et al. study [25] and 80.4% of patients in Azim's survey [26], 93.5% in Vosoughi et al.'s study [13] had low baseline serum 25(OH) vitamin D.

In our study, the mean SOFA and APACHEӀӀ scores (predictors of mortality and disease severity) and the mNUTRIC score were higher in the vitamin D deficient group than the group with normal serum vitamin D levels. This is consistent with the study by Anwar et al., which indicated the higher APACHE II score among the vitamin D-deficient group [27]. Another study also showed a higher SOFA score in the group with serum 25(OH)D levels of ≤ 10 ng/ml compared to 25(OH)D levels of > 10 ng/mL [28]. Furthermore, Aygencel et al. reported that the APACHE II and SOFA scores were higher in critically ill patients with vitamin D insufficiency [29]. A cohort study was conducted by Cecchi et al. to evaluate the correlation between vitamin D levels in septic patients and the clinical outcomes, and a clear correlation was reported between these variables [30].

Moreover, a prospective study conducted by Vosoughi et al. (2016) in Iran investigated the correlations between VDD, clinical outcomes, and mortality in 185 patients admitted to the ICU. The results demonstrated insufficient 25(OH)D levels in the patients. However, no significant correlation was observed between the 25(OH)D levels and clinical outcomes in the mentioned study [13].

According to Jeng et al., critical illness was associated with lower vitamin D levels in critically ill patients compared to healthy controls [31]. Similar to previous studies [21, 22, 32], our findings are in line with the result of Lee et al.[33], which indicated that VDD might increase mortality in patients admitted to the ICU. In addition, Moraes et al. suggested that low vitamin D levels upon ICU admission are an independent risk factor for mortality in critically ill patients[34]. In two separate systematic reviews and meta-analyses, Zhang et al.[3] (OR: 1.76; 95% CI: 1.38–2.24) and Hann et al.[35] (OR: 1.7; 95% CI: 1.49–2.16) reported that VDD is associated with the increased incidence of hospital mortality in critically ill adult patients. Meanwhile, Ralph et al. and Cecchi et al. reported no correlation between low vitamin D levels and increased mortality risk in critically ill patients [30, 36]. The meta-analysis conducted by Zhang et al. showed an association between VDD and mortality in patients admitted to the ICU[3]. In contrast, Haan et al. identified VDD as a risk factor for severe infections and mortality in critically ill patients [35].

A recent study of severe sepsis and septic shock patients indicated no correlation between VDD and 90-day mortality [29]. Although VDD may increase mortality in critically ill patients, the causes remain unknown and could be explained by several mechanisms. The higher mortality rate in critically ill patients with VDD may be due to the dysfunction of endothelial and immune cells and changes in glucose and calcium metabolism [37–39]. Endothelial cell dysfunction has been suggested as a potential cause of multiple organ dysfunction syndromes [40, 41]. In addition, VDD may intensify metabolic disorders and impair immune regulation, as reported in critically ill patients, leading to deteriorated clinical outcomes compared to patients with normal vitamin D levels. VDD may also increase the risk of inflammation by suppressing immune reactivity [42, 43]. Vitamin D improves immune responses against infections through the up-regulation of toll-like receptors (TLRs) [42].

Additionally, vitamin D has been reported to decrease the expression of pro-inflammatory cytokines [44] and increase the expression of anti-inflammatory cytokines [45]. Moreover, tissues in critically ill patients require more vitamin D, and VDD may lead to widespread tissue dysfunction in these cases. These mechanisms may clarify increased mortality and explain that it occurs due to systemic inflammatory response, organ failure, and metabolic dysfunction in critically ill patients.

In this study, patients with sufficient vitamin D levels experienced a shorter stay at the ICU. This finding is consistent with the McNally et al. study, where patients with low levels of Vitamin D stayed longer at the ICU and were later discharged compared to the sufficient group [46]. In addition, Zhang et al. and de Haan et al. reported the same results[3, 35]. On the contrary, Venkatram et al. and Aygencel et al. stated a significant relationship between low vitamin D levels and longer ICU stays. Therefore, it is suggested that the current findings could be due to the pleiotropic actions of vitamin D [29, 47].

In the present study, a negative association was observed between the length of mechanical ventilation and VDD in the patients admitted to the ICU. Consistent with our findings, a study conducted in India indicated that VDD increased the length of mechanical ventilation in critically ill patients [48]. Additionally, Quraishi et al. stated that plasma levels of 25(OH)D upon admission to the ICU were inversely associated with the duration of respiratory support [49]. In contrast to our findings, the observational, prospective study by Yaghoobi demonstrated no significant correlation between the length of mechanical ventilation and normal vitamin status in patients with ventilator-associated pneumonia and VDD [50]. Patients who require mechanical ventilation in the ICU often experience cellular changes and respiratory muscle weakness [51–54] that are exacerbated by factors such as malnutrition, electrolyte abnormalities, and severe infections [55]. Vitamin D is essential to human health, especially for bone and muscle function [56], while limited studies have investigated these effects on respiratory muscles.

This study had the main strength of a multicenter study with a large number of patients, which can improve the reliability of the results. However, several potential limitations should be addressed. First, this cross-sectional study cannot establish a cause-and-effect relationship. Second, some confounding factors, such as the type of disease that could bias the results, were not considered. In addition, the patient's serum level of vitamin D was measured only in the first 24 h of hospitalization, and the changes in the level of vitamin D during the hospitalization of the patients in the intensive care unit were not investigated. Prehospital health conditions such as the BMI influencing the results were not considered. Furthermore, more studies are needed to determine if there is a direct relationship between Vitamin D status and patient outcomes in ICU. For this, larger studies are needed to access reliable results.

## Conclusion

According to the results, the prevalence of VDD was high in critically ill patients admitted to the ICU. Moreover, inverse correlations were observed between vitamin D levels, disease severity (assessed by APACHE II and SOFA), and length of ICU stay. Importantly, VDD is the independent predictor of mortality in critically ill patients. Therefore, vitamin D administration may improve clinical outcomes in patients with VDD. However, more interventional studies are suggested to investigate the effect of vitamin D on clinical outcomes in patients admitted to the ICU.

### Supplementary Information

**Additional file 1: Table S1. **APCHE II (Acute Physiologic and Chronic Health Evaluation II) score content. **Table S2.** SOFA (Sequential Organ Failure Assessment) score content. **Table S3.** mNUTRIC (modified Nutrition Risk in Critically ill) score content.

## Data Availability

The datasets generated and analyzed during the current study and used for the preparation of the manuscript are included in the article submitted for publication.
