# Supplementary material for: Low plasma vitamin D is associated with increased 28-day mortality and worse clinical outcomes in critically ill patients
Source: BMC Nutr. 2024 Jan 9;10:6. doi: 10.1186/s40795-023-00801-1 (PMC10775546; doi:10.1186/s40795-023-00801-1)
Supplement: Supplementary file 1 — Additional file 1: Table S1. APCHE II (Acute Physiologic and Chronic Health Evaluation II) score content. Table S2. SOFA (Sequential Organ Failure Assessment) score content. Table S3. mNUTRIC (modified Nutrition Risk in Critically ill) score content. [file 40795_2023_801_MOESM1_ESM.docx]

**Legend of supplementary tables**

**Table S1** APCHE II (Acute Physiologic and Chronic Health Evaluation II) score content

**Table S2** SOFA (Sequential Organ Failure Assessment) score content

**Table S3** mNUTRIC (modified Nutrition Risk in Critically ill) score content

| **Table S1. APCHE II (Acute Physiologic and Chronic Health Evaluation II) score** | | | | | | | | | |
| --- | --- | --- | --- | --- | --- | --- | --- | --- | --- |
| High Abnormal Range Low Abnormal Range | | | | | | | | | |
| Physiologic Varible | +4 | +3 | +2 | +1 | 0 | +1 | +2 | +3 | +4 |
| Temperature | ≥41 | 39-40.9 |  | 38.5-38.9 | 36 -38.4 | 34-35.9 | 32-33.9 | 30-31.9 | <29.9 |
| Mean Arterial Pressure - mm Hg | >160 | 130-159 | 110-129 |  | 70-109 |  | 50-69 |  | <49 |
| HeartRate (ventricular response) | >180 | 140-179 | 110-139 |  | 70-109 |  | 55-69 | 40-54 | <39 |
| Respiratory Rate  (non-ventilated or ventilated) | >50 | 35-49 |  | 25-34 | 12-24 | 10-11 | 6-9 |  | <5 |
| Oxygen delivery (ml/min) or PaO_2_ (mm Hg) | >500 | 350-499 | 200-349 |  | <200   PO2>70 | PO2 61- 70 |  | PO2 55- 60 | PO2<55 |
| Arterial pH (preferred) | >7.7 | 7.6-7.69 |  | 7.5- 7.59 | 7.33- 7.49 |  | 7.25 -7.32 | 7.15 -7.24 | <7.15 |
| Serum HCO3 (venous mEq/l)  (not preferred, but may use if no ABGs) | >52 | 41-51.9 |  | 32-40.9 | 22-31.9 |  | 18-21.9 | 15-17.9 | <15 |
| Serum Sodium(mEq/l) | >180 | 160-179 | 155-159 | 150-154 | 130-149 |  | 120-129 | 111-119 | <110 |
| Serum Potassium(mEq/l) | >7 | 6 -6.9 |  | 5.5-5.9 | 3.5-5.4 | 3 -3.4 | 2.5 -2.9 |  | <2.5 |
| Serum Creatinine (mg/dl)  Double point score for acute renal failure | >3.5 | 2 -3.4 | 1.5 -1.9 |  | 0.6 - 1.4 |  | <0.6 |  |  |
| Hematocrit (%) | >60 |  | 50-59.9 | 46-49.9 | 30- 45.9 |  | 20 -29.9 |  | <20 |
| White Blood Count (total/mm3)  (in 1000s) | >40 |  | 20- 39.9 | 15 -19.9 | 3- 14.9 |  | 1- 2.9 |  | <1 |
| A. Total Acute Physiology Score (sum of 12 above points) | | | | | | | | | |
| B. Age points (years) <44=0; 45 to 54=2; 55 to 64=3; 65 to 74=5; >75=6 | | | | | | | | | |
| C. Chronic Health Points (see below) | | | | | | | | | |
| Total APACHE II Score (add together the points from A+B+C) | | | | | | | | | |

Chronic Health Points: If the patient has a history of severe organ system insufficiency or is immunocompromised as defined below, assign points as follows: 5 points for non-operative or emergency postoperative patients 2 points for elective postoperative patients

**Interpretation of Score:**

| Score | Death Rate (%) |
| --- | --- |
| 0-4 | 4 |
| 5-9 | 8 |
| 10-14 | 15 |
| 15-19 | 25 |
| 20-24 | 40 |
| 25-29 | 55 |
| 30-34 | 75 |
| >34 | 85 |

| **Table S2. SOFA (Sequential Organ Failure Assessment) score content** | | | | | |
| --- | --- | --- | --- | --- | --- |
| Organ System, Measurement | SOFA score | | | | |
|  | 0 | 1 | 2 | 3 | 4 |
| Respiration PaO_2_/FiO_2_, mmHg | Normal | <400 | <300 | <200  (with respiratory support) | <100  (with respiratory support) |
| Coagulation Platelets  x10^3^ /mm^3^ | Normal | <150 | <100 | <50 | <20 |
| Liver Bilirubin,mg/dL (µmol/l) | Normal | 1.2-1.9  (20-32) | 2.0-5.9 (33-101) | 6.0-11.9  (102-204) | >12.0  (<204) |
| Cardiovascular Hypotension | Normal | MAP<70  mmHg | Dopamine≤ 5 or dobutamine (any dose)** | Dopamine >5 or epinephrine ≤0.1 or norepinephrine ≤ 0.1 | Dopamine >15 or epinephrine > 0.1 or norepinephrine > 0.1 |
| Central Nervous System Glasgow Coma Score | Normal | 13-14 | 10-12 | 6-9 | <6 |
| Renal Creatinine, mg/dL (µmol/l) or  Urine output | Normal | 1.2-1.9 (110-170) | 2.0-3.4  (171-299) | 3.5-4.9  (300-440)  or <500 mL/day | >5.0  (>440)  or <200 mL/day |

**Interpretation of Score:**

| Maximum SOFA Score | Mortality |
| --- | --- |
| 0 to 6 | < 10% |
| 7 to 9 | 15 - 20% |
| 10 to 12 | 40 - 50% |
| 13 to 14 | 50 - 60% |
| 15 | > 80% |
| 15 to 24 | > 90% |

| **Table S3** mNUTRIC (modified Nutrition Risk in Critically ill) score content | | |
| --- | --- | --- |
| Variable Range Point | | |
| 0  1 | <50  50 to <75  ≥75 | Age |
| 0  1  2  3 | <15  15 to <20  20 to 28  ≥28 | APACHE II |
| 0  1  2 | <6  6 to <10  ≥10 | SOFA |
| 0  1 | 0 to 1  ≥2 | Number of comorbidities |
| 0  1 | 0 to <1  ≥1 | Days from hospital to ICU admission |

Interpretation of Score:

| Explanation | Category | Sum of points |
| --- | --- | --- |
| The patients have a high malnutrition risk | High score | 5-9 |
| The patients have a low malnutrition risk | Low score | 0-4 |
